# Therapy Processes Associated With Sudden Gains in Cognitive Therapy for Depression: Exploring Therapeutic Changes in the Sessions Surrounding the Gains

**DOI:** 10.3389/fpsyt.2021.576432

**Published:** 2021-03-23

**Authors:** Lotte H. J. M. Lemmens, Robert J. DeRubeis, Tony Z. Tang, Julia C. C. Schulte-Strathaus, Marcus J. H. Huibers

**Affiliations:** ^1^Department of Clinical Psychological Science, Faculty of Psychology and Neuroscience, Maastricht University, Maastricht, Netherlands; ^2^Department of Psychology, University of Pennsylvania, Philadelphia, PA, United States; ^3^Department of Clinical Psychology, Vrije Universiteit (VU) Amsterdam, Amsterdam, Netherlands; ^4^Empirically Validated Investment Strategies Co., San Juan, Puerto Rico

**Keywords:** cognitive therapy, major depression, sudden gains, mechanisms of change, time-course research

## Abstract

**Background:** The frequency and clinical impact of *Sudden Gains*—large symptom improvements during a single between-session interval—in psychotherapy for depression have been well established. However, there have been relatively few efforts to identify the processes that lead to sudden gains.

**Aim:** To explore therapy processes associated with sudden gains in cognitive therapy for depression by examining changes in the sessions surrounding the gains, and the session preceding the gain in particular.

**Methods:** Using ratings of video-recordings (*n* = 36), we assessed the content, frequency and magnitude of within-session cognitive-, behavioral-, and interpersonal change, as well as the quality of the therapeutic alliance in the session prior to the gain (pre-gain session), the session after the gain (post-gain session) and a control session. After that, we contrasted scores in the pre-gain session with those in the control session. In addition, we examined changes that occurred between the pre- and post-gain session (between-session changes) and explored patients' attributions of change.

**Results:** Although not statistically significant, within-session changes were more frequent and stronger in the pre-gain session compared to the control session. The largest difference between the pre-gain and control session was found in the behavioral domain, and reached the level of trend-significance. There were more, and more impactful between-session changes in the interval during which the gain occurred as compared to a control interval. Exploratory analysis of attributions of change revealed eight subcategories, all corresponding with the cognitive-, behavioral- and interpersonal- domain. The quality of the therapeutic alliance was high and almost identical in all sessions.

**Conclusion:** In spite of its small sample size, our study provides relevant descriptive information about potential precipitants of, themes related to, and attributions given for sudden gains. Furthermore, our study provides clear suggestions for future research. A better understanding of session content in the sessions surrounding sudden gains may provide insight into the mechanisms of change in psychotherapy, hereby suggesting treatment-enhancing strategies. We encourage researchers to conduct research that could clarify the nature of these mechanisms, and believe the methods used in this study could serve as a framework for further work in this area.

## Introduction

The frequency and clinical impact of *Sudden Gains* (1) in psychotherapy for depression have been well established [see meta-analyses of Aderka et al. (2) and Shalom and Aderka (3) for an overview]. Sudden gains, large symptom improvements during a single between-session interval, are observed in ~40% of depressed patients (range 25.9–50.0%), and those with sudden gains consistently report better acute and long-term treatment outcomes as compared to those without sudden gains (2, 3). Studies aimed at explaining why sudden gains occur have often focused on the predictive value of baseline characteristics (3). However, so far, no robust predictors of sudden gains have been identified, even in studies in which multiple predictors and their interactions were examined [e.g., (4)]. An explanation for this might lie in the strong association between sudden gains and treatment outcome, which suggests that this phenomenon is driven by important breakthroughs that occur during treatment that are difficult to predict using pre-treatment characteristics. One way to identify these breakthroughs is by meticulously analyzing the content of the sessions on either side of the gains, and the session preceding the gain (the so called pre-gain session) in particular. In only a few studies have researchers examined session content preceding sudden gains, and in those studies, the main focus has been on the role of cognitive change.

In the initial studies in cognitive therapy (CT), Tang and DeRubeis (1) and Tang et al. (5) examined the content of the pre-gain session and contrasted this to a control session. They found that the pre-gain session closely resembled the control session on most examined variables, including therapist competence and therapeutic alliance, but that there were differences in the cognitive domain. More specifically, they observed more cognitive change in the pre-gain session as compared to the control session, suggesting that cognitive changes might trigger sudden gains (1, 5). Researchers reporting on efforts to replicate this finding have concluded that cognitions were not related to sudden gains (6–9). It should be noted though, that Andrusyna et al. (7) examined this question in the context of psychodynamic therapy, and in the other studies, the role of cognition that was examined was substantially different than the one proposed and tested by Tang et al. For example, (6) tested the ability of a baseline self-report measure of cognition (prior to the initiation of a course of treatment) to predict which patients would experience sudden gains, and Kelly et al. (8) included a measure of self-reported self-esteem assessed at the beginning of the therapy session as a proxy of cognitive change, and associated this with sudden gains. Similarly, Vittengl et al. (9), amongst other methodological differences, also assessed process variables at the same point in time as they assessed depressive symptoms. Since in none of these studies the assessment of change was conducted in such a way to support, or rule out, the role of the purported mediator in the generation of a sudden gain, the relation between sudden gains and preceding cognitive changes still needs to be elucidated.

In addition, there is a growing body of research that collectively identifies sudden gains in a variety of other (non-cognitive) psychotherapeutic treatments for depression, or at a point in treatment in which cognitive techniques have not yet been addressed (7, 8, 10, 11). This suggests that other factors may be associated with sudden gains as well. Factors that have been suggested but that are lacking clear research support include, amongst others, behavioral- and interpersonal change, and the quality of the therapeutic alliance [e.g., (1, 6, 9, 12, 13)]. Additional research is necessary to examine the role of these factors more closely as well.

The current study focused on the identification of cognitive, behavioral, interpersonal, and relational precipitants of sudden gains in CT. Using the original studies by Tang and DeRubeis (1) and Tang et al. (5) as a starting point, video recordings of relevant sessions (pre-gain, post-gain and a control session) were watched and rated by independent raters, and the therapeutic changes that occurred in the pre-gain session (within-session change) were contrasted with observations obtained by viewing and rating control sessions. We also examined changes that occurred between the pre- and post-gain session (between-session change) and explored patients' attributions of change in the post-gain session. As such, we tried to identify crucial processes in and outside of therapy that might help us better understand how sudden gains occur. We expected that within-session changes would occur more frequently and with greater magnitude in the pre-gain session as compared to the control session, and that the most and most impactful between-session changes would be reported in the post-gain session. Because of the nature of CT we expected that most change would occur in the cognitive and the behavioral domain. However, because sudden gains have been found across psychotherapeutic interventions for depression, and at points in treatment in which cognitive techniques have not yet been addressed, we did not rule out that changes in the other domains could play a role as well.

## Methods

### Data Source

Data were collected by rating video recordings of relevant therapy sessions of 17 patients treated with CT who were identified as “*sudden gainers”* in a previous study in which we examined the frequency, magnitude, clinical impact and baseline predictors of sudden gains (14) and who gave consent for videotaping their sessions and for using these recordings for research purpose^1^. Participants were adult outpatients (nine women, mean age = 44.76 years; SD = 9.56) referred to the mood disorder unit of the Maastricht Academic Community Mental Health Centre in the Netherlands. All patients had received a primary diagnosis of major depressive disorder and 52.9% was diagnosed with recurrent depression. The majority of the patients (70.6%) was educated at intermediate vocational level (vs. 17.6% lower and 11.8% higher), and over half of the patients had a partner (58.8%) and was actively employed (52.9%) at the start of treatment. Baseline depression severity levels were assessed with the Beck Depression Inventory-II [BDI-II: (15)] and ranged from 17 to 54. The mean BDI-II score at baseline was 29.29 (SD = 9.96), which marks the border for “severe depression” (15). Treatment consisted of 16–20 individual 45-min sessions (mean = 15.76, SD = 4.10) and was based on the manual by Beck et al. (16). Sessions were offered weekly, but the protocol allowed flexibility in scheduling fewer appointments later on in treatment. The quality of therapy given was rated as very good to excellent and treatment dropout was low (17). The study is registered at the Netherlands Trial Register, part of the Dutch Cochrane Centre (ISRCTN 67561918). More details about the study design, participants, procedures, assessment instruments (quality of the), interventions and overall outcomes can be found in earlier publications (17–19).

**Figure 1:**
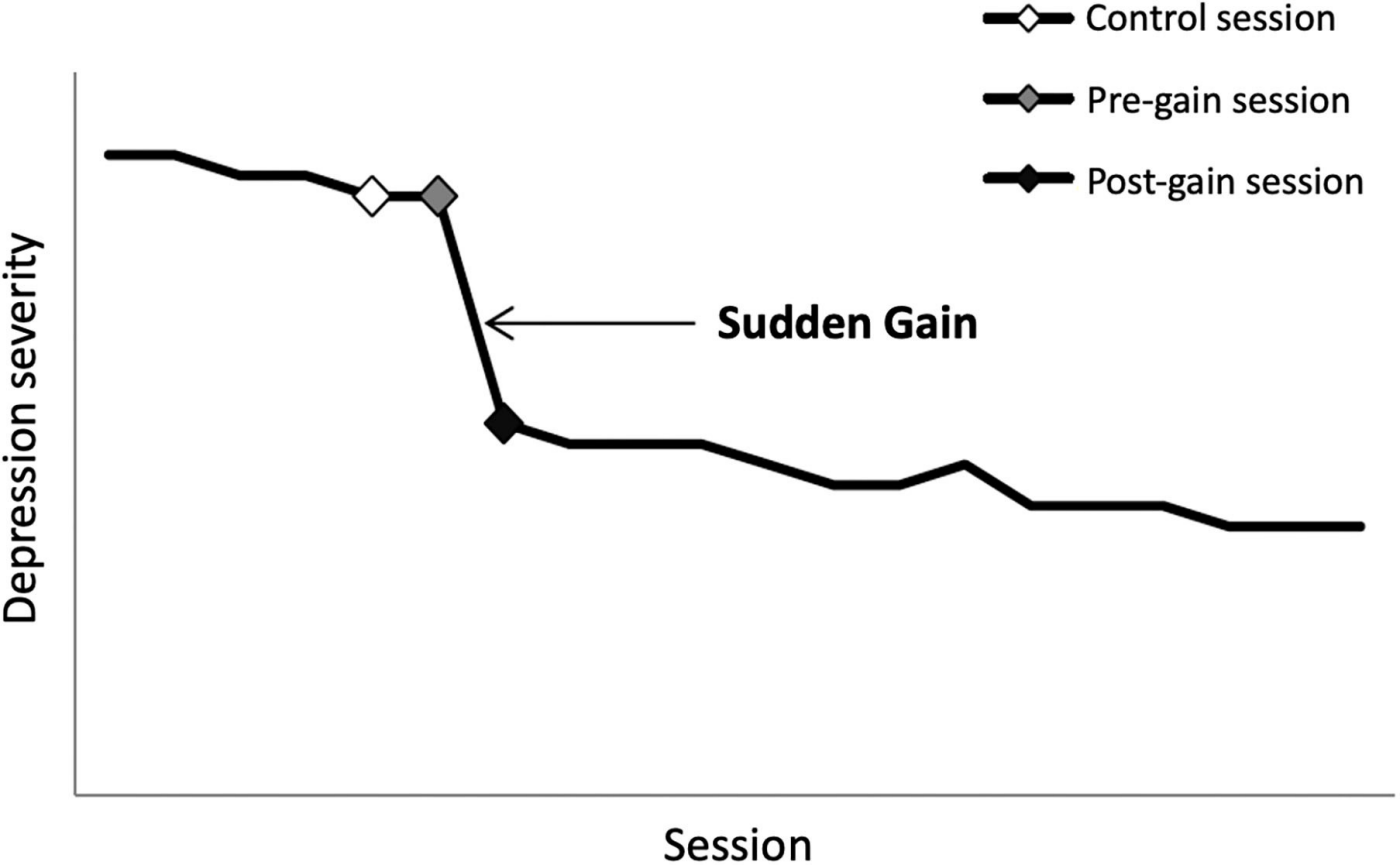
Illustration of the concept of sudden gains and the critical sessions surrounding the sudden gain. Note: the Y-axis represents depression severity with increasing severity from bottom to top; the X-axis represents session number with ascending session number from left to right. The black line represents the course of depression (simulated data; one data point per session). The large drop in symptoms between the gray and black diamond represents the sudden gain. The gray diamond represents the pre-gain session. The black diamond represents the post-gain session, and the white diamond represents the control session.

Sudden gains were examined using the *original* criteria as defined by Tang and DeRubeis [see (14) for more details]. Of the 17 patients included in the current study, 11 patients had one sudden gain and the other six experienced two. The average magnitude of the gains was 10.48 BDI-II points (SD = 4.12) and the median pre-gain session was session 6 (range 2–18). In order to collect data on processes that were hypothesized to be related to sudden gains, three sessions were selected for each patient, representing the session prior to the gain (pre-gain session), the session after the gain (post-gain session) and a within-subject control session. Following Tang and DeRubeis (1) and Tang et al. (5) we chose the session immediately before the pre-gain (the pre-pre-gain session) as the control session, because this session is most likely to resemble the pre-gain session (see Figure 1). For the six patients with two sudden gains, only the first gain was included, resulting in only one data point per patient [average magnitude (SD) of the gains = 10.59 (4.32) BDI-II points; median pre-gain session = 5]. Of the 51 selected sessions (3*17), recordings of 15 sessions were unavailable (missing or damaged), leaving 36 recordings available for analyses (12x pre-, 15x post-, and 9x control session). For eight patients, a full set of ratings was available, eight patients had data for the pre-gain and control session (same eight), and a total of 12 paired pre- and post-gain comparisons could be made.

### Measures

The frequency and magnitude of therapeutic changes in the pre-gain session, post-gain session and the control session were rated on three domains (1) the cognitive domain, (2) the behavioral domain, and (3) the interpersonal domain. In addition, the quality of the therapeutic alliance in each session was assessed. Cognitive-, behavioral- and interpersonal- changes were assessed using an adapted and extended version of the Patient Cognitive Change Scale [PCCS; (1, 5)] that was composed for the current study^2^. We included nine items of the original PCCS: six items assessing potential cognitive change, and three items reflecting behavioral change. Unlike the original studies, in which all nine items were fitted in the cognitive domain, we created a separate behavioral domain, including two additional items of the Activation subscale of the Behavioral Activation for Depression Scale [BADS; (20)]. Furthermore, following the structure of the cognitive items of the PCCS, we created comparable items for the interpersonal domain using the IPT manual by Klerman et al. (21). An overview of the items used can be found in Table 1. The complete instrument including its rating instructions can be found in Supplementary Data I. Items reflected either preparation for change (indicated with an ^*^ in Table 1) or actual change achieved. For each item, raters first indicated whether such change was observed in the session (frequency rating; yes/no). If this was the case, they specified its content and indicated whether the (preparation for) change was achieved during the session (within-session change) or whether it reflected a discussion of change that occurred prior to the session (between-session change). After that, following the original guidelines from the PCCS, the personal significance of the change (magnitude rating) was rated on a five-point scale ranging from 0 (no change/not applicable) to 4 (change with extraordinary personal significance). Any of the items could receive multiple ratings in a given session, as long as they clearly differed in content. However, if the same type of progress was acknowledged more than once, only one score was given. Raters were instructed never to infer changes. Only when any of the changes were explicitly acknowledged, raters would classify it by its content and rate its magnitude. Total scores per domain (separate sum scores for frequency and magnitude ratings) were obtained by summing up all item scores per category.

**Table 1:** Overview of items for the cognitive, behavioral and interpersonal domain.

**Domain**	**Items**
Cognitive change	1. The patient became aware of the relationship between cognition and mood^*^ 2. The patient became aware of a belief behind negative feelings^*^ 3. The patient changed his/her belief 4. The patient became aware of schema^*^ 5. The patient changed his/her schema 6. The patient accepted a new cognitive technique^*^
Behavioral change	1. The patient accepted alternative behavior^*^ 2. The patient decided to increase pleasurable activities^*^ 3. The patients made plans for pleasurable activities^*^ 4. The patient engaged in a wide and diverse array of activities 5. The patient structured his/her day's activities
Interpersonal change	1. The patient became aware of the relation between interpersonal functioning and mood^*^ 2. The patient became aware of dysfunctional patterns in interpersonal functioning^*^ 3. The patient became aware of the need to improve interpersonal functioning^*^ 4. The patient decided to change interpersonal functioning^*^ 5. The patient made plans for changing interpersonal functioning^*^ 6. The patient changed interpersonal functioning

* *Items reflect preparation for change (vs. actual change achieved). For each item, raters first indicated whether such change was observed in the session (frequency rating; yes/no). If this was the case, they specified its content and indicated whether the (preparation for) change was achieved during the session (within-session change) or whether it reflected a discussion of change that occurred prior to the session (between-session change). After that, the significance of the change (magnitude rating) was rated on a five-point scale (0 = no change/item not applicable; 1 = possible/potential change; 2 = definite change; 3 = important change; 4 = change with extraordinary personal significance)*.

The quality of the therapeutic alliance was assessed with the observer-rated version of the Working Alliance Inventory Short [WAI-O-S; (22, 23)], which is based on Bordin's (24) conceptualization of working alliance. According to Bordin, a strong alliance forms if the therapist and client agree on (1) the *goals* of therapy and (2) the *tasks* that are needed to meet those goals, and (3) have a *bond* between them that facilitates this process. The instrument consists of 12 items (four for each subscale) that are rated on a 7-point scale (1 = never, to 7 = always), with higher scores indicating a stronger alliance. A total score is obtained by summing up all item scores. Psychometric properties of the WAI-O-S are good (7, 25, 26).

Patients' attributions for change in the post-gain session were explored using a rater-based modified version of the Symptom Change Attribution Interview [SCAI; (27)]. Raters indicated whether (1) there was a discussion between therapist and client about an improvement in mood (yes/no); (2) whether the patient reported reasons for change, and if so, which reason(s) was/were reported; (3) whether the patient report on anything from the previous session that stood out for him/her, and if so, what stood out; and (4) whether the patient reported that during/since the last session (s)he (a) realized something not realized before, arrived at new perspective on something, or changed beliefs or ideas, (b) learned new techniques that (s)he found helpful, (c) learned other things, or (d) has noticed that (s)he has been doing anything different, plus specification.

### Procedure

Each session was watched and rated by two independent raters under the supervision of the first author (LL) who was trained by TT and RD. Raters were clinical psychology students (1 undergraduate, 1 MSc) from Maastricht University (Netherlands). Individual scores on all items were discussed afterwards with the first author until consensus was reached. Consensus scores were used as the final scores. Prior to the study, raters individually orientated on the topic by means of an extensive literature search (8 h). In addition, raters received an elaborate training (9 × 2 h) in which they were taught about the concept of sudden gains, the instrument, the rating guidelines, and the complexities of rating psychotherapeutic processes. Throughout the 3 month rating period, weekly consensus meetings were scheduled to optimize reliability and minimize rater's drift. In each session, a subset of tapes was discussed, and the conclusions from each session were implemented in the strategy for the next subset of tapes. All identifying information was removed from the recordings to make sure that all of those involved in the rating process were blind for the session number, symptom changes before and after the session, and treatment outcome. Due to the specific therapeutic interventions and the visual character of the study, it was not possible to blind raters for patients and therapist. Whenever raters heard that change was explicitly acknowledged in the session, they were asked to specify this as clearly as possible and to provide their line of reasoning for selecting a certain magnitude rating. This was important for both the training phase as well as the rating phase as this facilitated the consensus discussions. There were no written transcripts available. Instead raters were instructed to press pause and/or rewind the recording if necessary.

### Data Analysis

We replicated and extended the method used by Tang and DeRubeis (1) and Tang et al. (5). First, using all available data of all 17 patients, we mapped out the frequency and magnitude (mean, SD) of within-session and between-session changes that were observed in the relevant sessions. After that, for those with complete data, frequency and magnitude of within-session changes in each domain were compared between the pre-gain session and the control session using paired samples *t*-tests (*n* = 8). Similar to the original studies, if the frequency or magnitude of an observed variable in the pre-gain session was significantly (*p* < 0.05) higher than its level in the control session, we considered this a suggestion that this factor might be associated with the sudden gain. To gain insight in important changes that happened between the pre- and post-gain session, we took a closer look at the between-session ratings in the post-gain session, and contrasted them to the scores in the pre-gain session using paired-samples *t*-test (paired data only; *n* = 12) Finally, we explored and manually categorized the content of the attribution questions in the post-gain session, to learn more about patients' own attributions of change (see Supplementary Data I for more details).

## Results

### Data Exploration

An overview of all available within- and between-session changes in each of the three domains (frequency and magnitude ratings) and alliance scores (M, SD) in the pre-gain, post-gain and control session are presented in Table 2. A total of 103 within-session changes were identified; 44 in the pre-gain, 40 in the post-gain, and 19 in the control session. Although in general, magnitude ratings were in the lower end of the range, the largest magnitude ratings were observed in the pre-gain sessions, with highest overall domain scores in the behavioral domain, followed by the interpersonal and cognitive domain. The control sessions showed the smallest overall within-session change. There were five items that were not observed in any of the sessions. For three of them (engaging in a wide and diverse array of activities, structuring activities, and changing interpersonal functioning), we did not expect within-session changes, since they require action outside of the session. For the other two items (becoming aware of/changing a schema), within-session change was possible, but did not occur. The total number of between-session changes that was observed was 162; 54 for the pre-gain session, 70 for the post-gain session, and 38 for the control session. Largest magnitude ratings were observed in the post-gain session, with a similar pattern for the various domains as was found in the within-session ratings (i.e., highest overall domain score in the behavioral domain, followed by interpersonal and cognitive domain). All items were rated at least once in any of the sessions, except for the cognitive items becoming aware of and/or changing a schema, which were not observed at all. The quality of the therapeutic alliance was high and almost identical in all sessions.

**Table 2:** Overview of all available data: within- and between-session cognitive, behavioral and interpersonal change (frequency and magnitude) and within-session alliance data (M, SD) in the pre-gain, post-gain and control session.

**(*n* = 17)**	**Within-session change**						Between-session change^**^					
	**Pre-gain session** (***n*** **= 12)**		**Post-gain session** (***n*** **= 15)**		**Control session** (***n*** **= 9)**		**Pre-gain session** (***n*** **= 12)**		**Post-gain session** (***n*** **= 15)**		**Control session** (***n*** **= 9)**	
**Domain**	**Frequency** **(Sum)**	**Magnitude** **(M, SD)**	**Frequency** **(Sum)**	**Magnitude** **(M, SD)**	**Frequency** **(Sum)**	**Magnitude** **(M, SD)**	**Frequency** **(Sum)**	**Magnitude** **(M, SD)**	**Frequency** **(Sum)**	**Magnitude** **(M, SD)**	**Frequency** **(Sum)**	**Magnitude** **(M, SD)**
**Cognitive domain total**	**13**	**2.17 (2.66)**	**16**	**2.07 (2.40)**	**9**	**1.56 (1.33)**	**12**	**1.42 (2.50)**	**12**	**1.80 (2.73)**	**3**	**0.89 (1.36)**
- Becoming aware of relation cognition and mood^*^	1	0.08 (0.29)	4	0.53 (1.13)	3	0.33 (0.50)	3	0.33 (0.65)	2	0.20 (0.56)	0	0.00 (0.00)
- Becoming aware of belief^*^	3	0.50 (0.90)	3	0.33(0.72)	2	0.44 (0.88)	3	0.25 (0.45)	5	0.67 (1.11)	1	0.33 (1.00)
- Changing a belief	6	1.08 (1.56)	3	0.47 (1.06)	0	0.00 (0.00)	3	0.42 (1.44)	2	0.40 (1.06)	1	0.33 (1.00)
- Becoming aware of schema^*^	0	0.00 (0.00)	0	0.00 (0.00)	0	0.00 (0.00)	0	0.00 (0.00)	0	0.00 (0.00)	0	0.00 (0.00)
- Changing a schema	0	0.00 (0.00)	0	0.00 (0.00)	0	0.00 (0.00)	0	0.00 (0.00)	0	0.00 (0.00)	0	0.00 (0.00)
- Accepting a new cognitive technique^*^	3	0.50 (0.90)	6	0.73 (1.28)	4	0.78 (1.09)	3	0.42 (1.16)	3	0.53 (1.13)	1	0.22 (0.67)
**Behavioral domain total**	**17**	**2.67 (3.28)**	**5**	**0.60 (1.59)**	**1**	**0.22 (0.67)**	**23**	**3.08 (2.91)**	**36**	**5.07 (4.06)**	**20**	**4.22 (4.27)**
- Accepting alternative behavior^*^	7	1.25 (1.66)	3	0.33 (0.72)	1	0.22 (0.67)	7	0.92 (1.08)	10	1.53 (1.41)	9	1.89 (1.83)
- Deciding to increase pleasurable activities^*^	5	0.75 (0.97)	1	0.13 (0.52)	0	0.00 (0.00)	3	0.33 (0.65)	7	1.00 (1.13)	2	0.44 (0.88)
- Making plans for pleasurable activities^*^	5	0.67 (1.07)	1	0.13 (0.52)	0	0.00 (0.00)	4	0.42 (0.67)	7	1.00 (1.20)	3	0.56 (0.88)
- Engaging in a wide and diverse array of activities	0	0.00 (0.00)	0	0.00 (0.00)	0	0.00 (0.00)	5	0.83 (1.11)	10	1.40 (1.18)	3	0.78 (1.30)
- Structured day's activities	0	0.00 (0.00)	0	0.00 (0.00)	0	0.00 (0.00)	4	0.58 (1.00)	2	0.13 (0.35)	3	0.56 (1.01)
**Interpersonal domain total**	**14**	**2.33 (3.98)**	**19**	**2.33 (3.75)**	**9**	**1.67 (2.35)**	**19**	**2.50 (3.34)**	**22**	**3.33 (4.88)**	**15**	**3.00 (2.87)**
- Becoming aware of relation int. func. and mood^*^	1	0.17 (0.58)	3	0.27 (0.59)	0	0.00 (0.00)	4	0.67 (1.15)	6	0.93 (1.28)	5	1.11 (1.17)
- Becoming aware of dysfun. patterns in int. func^*^	3	0.50 (1.00)	5	0.73 (1.10)	3	0.44 (0.73)	5	0.75 (1.06)	3	0.53 (1.13)	5	1.00 (1.00)
- Becoming aware of need to improve int. func^*^	3	0.50 (1.00)	4	0.53 (0.92)	2	0.33 (0.71)	3	0.33(0.65)	3	0.33 (0.72)	2	0.33 (0.71)
- Deciding to change interpersonal functioning^*^	3	0.58 (1.00)	4	0.47 (0.92)	2	0.44 (0.88)	2	0.33 (0.78)	2	0.33 (0.90)	1	0.11 (0.33)
- Making plans interpersonal change^*^	4	0.58 (1.08)	3	0.33 (0.90)	2	0.44 (0.88)	1	0.08 (0.29)	4	0.53 (1.13)	1	0.22 (0.67)
- Changed interpersonal functioning	0	0.00 (0.00)	0	0.00 (0.00)	0	0.00 (0.00)	4	0.33 (0.49)	4	0.67 (1.40)	1	0.22 (0.67)
**Therapeutic alliance total**		**66.83 (2.86)**		**66.60 (2.23)**		**65.89 (4.51)**						
- Bond		21.92 (1.51)		21.60 (1.18)		22.11 (2.03)						
- Goal		23.00 (0.74)		22.93 (0.88)		22.78 (1.09)						
- Task		22.25 (1.14)		22.53 (1.13)		21.22 (1.79)						

* *Items reflect preparation for change (vs. actual change achieved). For each item, raters first indicated whether such change was observed in the session (yes/no; frequency rating). If this was the case, they specified its content and indicated whether the (preparation for) change was achieved during the session (within-session change) or whether it reflected a discussion of change that occurred prior to the session (between-session change). After that, the significance of the change (magnitude rating) was rated on a five-point scale (0 = no change/item not applicable; 1 = possible/potential change; 2 = definite change; 3 = important change; 4 = change with extraordinary personal significance)*.

** *since alliance was only rated within the session, there is no between-session alliance data available*.

### Within-Session Changes

Table 3 presents frequency and magnitude ratings (M, SD) of within session changes on each of the three domains in the pre-gain and control session for those with complete data (*n* = 8). Furthermore, alliance scores (M, SD) for these sessions are presented. There were more within-session changes in the pre-gain session as compared to the control session (23 vs. 16 for pre-gain and control session, respectively). Magnitude scores in the cognitive-, behavioral- and interpersonal domain were larger in the pre-gain session as compared to the control session. The largest difference between the pre-gain and the control session was found in the behavioral domain, followed by the cognitive and the interpersonal domain. Paired-samples *t*-tests indicated that none of these differences were statistically significant, but the difference in the behavioral items reached trend level (*p* = 0.09). The average magnitude rating of behavioral change in pre-gain session (2.0) was equivalent to one “definite change” in behavioral items, whereas the average control session (0.25) represented almost no change in behavioral items.

**Table 3:** Within-session cognitive, behavioral and interpersonal change (frequency and magnitude) and alliance data (M, SD) in the pre-gain and control session, and their comparison (*n* = 8).

	**Within session change**				
	**Pre-gain session**		**Control session**		
**Domain**	**Frequency** **(Sum)**	**Magnitude** **(M, SD)**	**Frequency** **(Sum)**	**Magnitude** **(M, SD)**	**Pre vs. control** **(*p*)**
**Cognitive domain total**	**7**	**1.63 (2.50)**	**6**	**1.25 (1.04)**	**0.76**
- Becoming aware of relation cognition and mood^*^	1	0.13 (0.35)	2	0.25 (0.46)	0.60
- Becoming aware of belief^*^	2	0.50 (0.93)	1	0.25 (0.71)	0.60
- Changing a belief	2	0.50 (0.93)	0	0.00 (0.00)	0.17
- Becoming aware of schema^*^	0	0.00 (0.00)	0	0.00 (0.00)	1.00
- Changing a schema	0	0.00 (0.00)	0	0.00 (0.00)	1.00
- Accepting a new cognitive technique^*^	2	0.50 (0.93)	3	0.75 (1.16)	0.70
**Behavioral domain total**	**8**	**2.00 (3.07)**	**1**	**0.25 (0.71)**	**0.09**
- Accepting alternative behavior^*^	4	1.13 (1.64)	1	0.25 (0.71)	0.09
- Deciding to increase pleasurable activities^*^	2	0.50 (0.93)	0	0.00 (0.00)	0.17
- Making plans for pleasurable activities^*^	2	0.38 (0.74)	0	0.00 (0.00)	0.20
- Engaging in a wide and diverse array of activities	0	0.00 (0.00)	0	0.00 (0.00)	1.00
- Structured day's activities	0	0.00 (0.00)	0	0.00 (0.00)	1.00
**Interpersonal domain total**	**8**	**2.00 (3.51)**	**9**	**1.88 (2.42)**	**0.82**
- Becoming aware of relation int. func. and mood^*^	0	0.00 (0.00)	0	0.00 (0.00)	1.00
- Becoming aware of dysfun. patterns in int. func^*^	2	0.38 (0.74)	3	0.50 (0.76)	0.60
- Becoming aware of need to improve int. func^*^	2	0.38 (0.74)	2	0.38 (0.74)	1.00
- Deciding to change interpersonal functioning^*^	2	0.63 (1.19)	2	0.50 (0.93)	0.35
- Making plans interpersonal change^*^	2	0.63 (1.19)	2	0.50 (0.93)	0.35
- Changed interpersonal functioning	0	0.00 (0.00)	0	0.00 (0.00)	1.00
**Therapeutic alliance total**		**66.25 (3.24)**		**66.63 (4.21)**	**0.75**
- Bond		21.88 (1.81)		22.38 (2.00)	0.47
- Goal		22.75 (0.71)		23.00 (0.93)	0.52
- Task		21.88 (1.13)		21.50 (1.69)	0.50

* *Items reflect preparation for change (vs. actual change achieved). For each item, raters first indicated whether such change was observed in the session (yes/no; frequency rating). If this was the case, they specified its content and indicated whether the (preparation for) change was achieved during the session (within-session change) or whether it reflected a discussion of change that occurred prior to the session (between-session change). After that, the significance of the change (magnitude rating) was rated on a five-point scale (0 = no change/item not applicable; 1 = possible/potential change; 2 = definite change; 3 = important change; 4 = change with extraordinary personal significance)*.

A closer look at the item level, indicated that the effects in the behavioral domain seemed mainly driven by within-session acceptance of new behavior, the decision to increase pleasurable activities, and making plans for these pleasurable activities. All of these items represented preparation for change. The largest contrast reflecting actual change achieved in the session was found in the cognitive domain (changing a belief). The quality of therapeutic alliance was similar in the pre-gain and control session.

### Between-Session Changes

Table 4 presents the frequency and magnitude ratings (M, SD) of between-session changes, i.e., changes that were discussed in the session but that occurred outside the therapy, on each of the three domains between the two session in which the gain occurred for patients with complete data in these sessions (*n* = 12). As can be seen in the table, more (frequency rating) and more impactful (magnitude rating) between-session changes were observed in the post-gain sessions as compared to the pre-gain session. For the behavioral domain, this difference was at the level of a non-significant trend (overall domain score *p* = 0.07). At the item-level, patients accepted significantly more alternative behavior (*p* = 0.03). Furthermore, patients reported more plans for pleasurable activities, and engaged in a wider and more diverse array of activities between the two sessions, at the level of a non-significant trend (*p* = 0.09 and *p* = 0.10, respectively).

**Table 4:** Between-session cognitive, behavioral and interpersonal change (frequency and magnitude) in the pre-gain and post-gain session, and their comparison (*n* = 12).

	**Prior to session change**				
	**Pre-gain session**		**Post-gain session**		
**Domain**	**Frequency** **(Sum)**	**Magnitude** **(M, SD)**	**Frequency** **(Sum)**	**Magnitude** **(M, SD)**	**Pre vs. post** **(*p*)**
**Cognitive domain total**	**12**	**1.42 (2.50)**	**12**	**2.25 (2.90)**	**0.36**
- Becoming aware of relation cognition and mood^*^	3	0.33 (0.65)	2	0.25 (0.62)	0.78
- Becoming aware of belief^*^	3	0.25 (0.45)	5	0.83 (1.19)	0.13
- Changing a belief	3	0.42 (1.44)	2	0.50 (1.17)	0.79
- Becoming aware of schema^*^	0	0.00 (0.00)	0	0.00 (0.00)	1.00
- Changing a schema	0	0.00 (0.00)	0	0.00 (0.00)	1.00
- Accepting a new cognitive technique^*^	3	0.42 (1.16)	3	0.67 (1.23)	0.46
**Behavioral domain total**	**23**	**3.08 (2.91)**	**33**	**5.92 (4.03)**	**0.07**
- Accepting alternative behavior^*^	7	0.92 (1.08)	9	1.75 (1.42)	0.03
- Deciding to increase pleasurable activities^*^	3	0.33 (0.65)	7	1.25 (1.14)	0.05
- Making plans for pleasurable activities^*^	4	0.42 (0.67)	7	1.25 (1.22)	0.09
- Engaging in a wide and diverse array of activities	5	0.83 (1.11)	8	1.50 (1.24)	0.10
- Structured day's activities	4	0.58 (1.00)	2	0.17 (0.39)	0.21
**Interpersonal domain total**	**19**	**2.50 (3.34)**	**19**	**3.58 (5.37)**	**0.37**
- Becoming aware of relation int. func. and mood^*^	4	0.67 (1.15)	5	1.00 (1.35)	0.34
- Becoming aware of dysfun. patterns in int. func^*^	5	0.75 (1.06)	2	0.42 (1.00)	0.17
- Becoming aware of need to improve int. func^*^	3	0.33 (0.65)	2	0.25 (0.62)	0.72
- Deciding to change interpersonal functioning^*^	2	0.33 (0.78)	2	0.42 (1.00)	0.84
- Making plans interpersonal change^*^	1	0.08 (0.29)	4	0.67 (1.23)	0.15
- Changed interpersonal functioning	4	0.33 (0.49)	4	0.83 (1.53)	0.24

* *Items reflect preparation for change (vs. actual change achieved). For each item, raters first indicated whether such an change was observed in the session (yes/no; frequency rating). If this was the case, they specified its content and indicated whether the (preparation for) change was achieved during the session (within-session change) or whether it reflected a discussion of change that occurred prior to the session (between-session change). After that, the significance of the change (magnitude rating) was rated on a five-point scale (0 = no change/item not applicable; 1 = possible/potential change; 2 = definite change; 3 = important change; 4 = change with extraordinary personal significance)*.

### Patients' Attributions for Change

In all 12 post-gain sessions, there was a spontaneous discussion about improvements in mood that occurred prior to the session. In all but one of the sessions, the patient reported one or more reasons for symptom improvement; eight patients reported that they realized something they had not realized before, and/or arrived at a new perspective or changed ideas/beliefs during/since the last session; seven patients indicated that they learned something that they found helpful; and five patients reported noticing themselves doing things differently. Attributions could be sorted into the following:

Eight subcategories, all corresponding with the cognitive-, behavioral- and interpersonal- domain: behavioral activation, exercise, shift in belief(s)/perspective(s), absence of negative thoughts, work/work-life balance, asking for help, setting and communicating boundaries and other (see Table 5 for examples). Two patients explicitly linked their improvement to the previous session (“*I realized last session that I don't know if my negative thoughts will influence my future”* and “*I realized last week that my depression worsens if I stay inactive. I have to get up and do things*.”

**Table 5:** Patients' attributions for change as reported in the post-gain session (categories and examples) (*n* = 12).

**Category**	**Examples**
- Behavioral activation	Had more things to do this week (mentioned by two patients); Did a lot of pleasurable things; Took up old activities; Went shopping; Got up and did things.
- Exercise	Started going to the gym.
- Shift in belief/perspective	Future is brighter than expected; It's not my fault; I cannot know if negative thoughts will influence future.
- Absence of negative thoughts	Had no negative thoughts this week.
- Work and work-life balance	Things are going well at work; Job application going well; Getting used to combination work/private life.
- Asking for help	Asked husband to make coffee; Asked husband to help with laundry.
- Setting/communicating boundaries	Has set clearer boundaries; Has set new rules and communicated them to partner.
- Other	Had a nice holiday.

## Discussion

The present study explored therapy processes associated with sudden gains in CT for depression by examining the role of cognitive, behavioral, and interpersonal change and the quality of the therapeutic alliance in the sessions surrounding the gains. More specifically, using ratings of video-recordings, we assessed the content, frequency and magnitude of within-session changes in each of the three domains, and the quality of the therapeutic alliance in the session prior to the gain (pre-gain session), the session after the gain (post-gain session) and a control session. After that, we contrasted scores in the pre-gain session with those in the control session. In addition, we examined changes that occurred between the pre- and post-gain session (between-session changes) and explored patients' attributions of change in the post-gain session.

Although the sample size was small, absolute magnitude scores were in the lower end of the range, and several constructs were not observed at all, within-session changes were observed more frequently and with greater magnitude in the pre-gain session as compared to the control session, albeit not statistically significant. The largest difference between the pre-gain and the control session was found in the behavioral domain, and reached the level of trend-significance, which is interesting and in line with a C(B)T context. Other within-session changes in the pre-gain sessions were mainly preparation for change (i.e., awareness, openness, realizations). The most promising item that reflected actual change achieved during the pre-gain session was the item “change of belief,” which also matches a C(B)T setting. Although not statistically significant, possibly because of the limited statistical power, our pattern of findings is similar to those reported by Tang and DeRubeis (1) and Tang et al. (5).

The fact that independent raters observed more between-session as compared to within-session changes was not totally unexpected given that several items (those reflecting actual change) could not be rated as within-session change since they required action outside of the therapist's office. For between-session change, all items could potentially be rated. Furthermore, the period for assessing between-session changes (a full between-session interval) was a lot longer than that for within-session change (a 45-min session), which allowed for more opportunities for change. It should be noted that this also increases chances of recall bias. The frequency and impact of between-session changes were larger for the post-gain session as compared to the pre-gain session. Although not statistically significant, this indicated the changes in the between-session interval in which the gain occurred were more frequent and stronger as compared to those in the control interval. Rudimentary analysis of attributions of change revealed eight subcategories of explanations for change, all corresponding to each of the three investigated domains. The quality of the therapeutic alliance was high and almost identical in all sessions.

### Scores in the Cognitive, Behavioral and Interpersonal Domain

Although consistent with previous studies conducted by Tang and DeRubeis (1), Tang et al. (5), and, scores in the various domains were on the lower end of the range. More specifically, even though the magnitude scale ranged from 0 to 4, ratings higher than 2 were rarely given. The question that remains is why this was the case. Though speculative, some reasons are more plausible than others and deserve further discussion. First, our findings could indicate that treatment was not powerful enough to elicit important changes in the examined domains. However, this explanation is unlikely since an extensive integrity check confirmed high therapy quality and integrity [see (17)]. It is more likely that magnitude of change in this study is underrepresented because of the specific rating instructions that were used. Similar to Tang and DeRubeis (1) and Tang et al. (5), raters were instructed not to infer, but rather only rate change that was explicitly acknowledged in the session. As a result, changes that were more implicit were not detected, despite potentially contributing to change. A third reason for the relatively low ratings might lie in the scale that was used for the magnitude scores. The most information seemed to be in the differentiation between 0 (no change) and 2 (definite change). The fact that scores higher than two were rarely given, indicates that it is difficult—or even unnecessary—to further differentiate between 2 (definite change), 3 (important change) and 4 (change with extraordinary personal significance). Alternatively, our findings could point toward the possibility that the domains that were examined in this study were not important for sudden gains, but that other therapy processes that were not investigated in this study are crucial for these large and sudden drops in depressive symptoms.

When taking a closer look at the specific domains that were included, particularly the scores in the cognitive domain were lower than expected. In fact, items related to identifying and changing schemas were not even rated at all (either as within- or between-session change). This is remarkable when taking into account that the cognitive domain is a central part of CT. Although the possibility that cognitive changes are not important for sudden gains in CT cannot be fully ruled out, it is more likely that behavioral and interpersonal changes were easier to detect. One reason for this might be that therapists relied more on behavioral techniques instead cognitive techniques within the therapeutic framework, or that schema change is very difficult to track by raters. Since the independent raters were instructed not to infer, they had to follow the discussion as it emerged during the session. The difference between the domains might be further reinforced by the fact that, contrary to several other studies, we combined the various domains in one study. During the rating process, we noticed that several examples would fit in multiple domains (e.g., cognitive change and interpersonal change). In our case, we had to decide which category fitted best. Moreover, in a way, one could even argue that in well-delivered CT, all behavioral/interpersonal change follows from or leads to cognitive change. Unfortunately, our research design did not allow us to further differentiate here.

A final potential explanation that cannot remain undiscussed is the fact that with our approach we made a critical assumption: namely that explicit changes in the pre-gain session (either in terms of preparation for change, or actual change) are responsible for the gains. Although this framework is more plausible than for instance the idea that sudden gains are predicted by baseline levels of hypothesized therapeutic processes, it gives a very central position to the session itself and rules out various other options, such as the possibility that during the pre-gain session a “seed” is planted that is followed-up later on during the week, or that sudden gains are not linked to the therapy sessions at all. If this would be the case, it is not remarkable that we only observed little changes. Although we tried to shed light on this by also looking at changes that occurred in the between-session interval in which the gain occurred, and by exploring the patients' own attributions for change, we did not do this systematically enough to provide clear cut answers about this. In order to get a clearer view about this one would need more fine-grained research on the patients' lives outside of the session, as well as more structured information about attributions of change. Furthermore, it would be interesting to differentiate between procedures (interventions used by the therapist) and processes [changes experienced by the patient, presumably following from procedures; (28)]. Because if the therapist uses more specific techniques in the session prior to the gain, this could also inform us about important precipitants of sudden gains.

### The Role of Therapeutic Alliance

Contrary to the rather low scores in the behavioral, cognitive and interpersonal domains, alliance scores were very high and almost identical in every session. What does that tell us about the role of alliance in sudden gains? The fact that there were no differences between nor changes during the sessions makes it unlikely that alliance is a process that drives sudden gains. However, the high scores might point toward alliance as a prerequisite for sudden gains. Contrasting the quality of the therapeutic alliance between those with and without sudden gains could shed more light on this. Unfortunately, those data were not available. Alternatively, as suggested by Zilcha-Mano et al. (4), it could also point toward alliance as an important ingredient of an upward spiral in which sudden gains lead to a further strengthening in alliance, which in turn predict further improvements in well-being, which in may result in sustained sudden gains.

### Methodological Considerations

To our knowledge, our study was the first to test and replicate the original hypothesis regarding cognitive change proposed by Tang and DeRubeis (1) and Tang et al. (5). Furthermore, we extended the work of Tang et al. by examining the role of changes in the behavioral and interpersonal domain as well. Other strengths of the study include the line-by-line analysis of video recordings by independent raters, exploration of changes that occurred in the between-session interval in which the gain occurred and the inclusion of a rudimentary attribution analysis. However, several limitations should be mentioned as well. First, our study was a secondary analysis of an existing trial that was not specifically designed to answer questions about precipitants of sudden gains. Therefore, our design also had some restrictions. For example, there was no systematic attribution interview. Furthermore, a systematic and fine-grained analysis of changes that happened in-between sessions was lacking and we cannot completely guarantee that all between-session changes and that that were discussed actually happened in the single between-session interval that was examined. Although CT is a present-focused treatment by nature, and therapists were instructed to specifically ask for change since the last session, there were instances for which the interval was not made explicit. In addition, although our instrument was largely composed from existing and validated instruments, the psychometric properties of the instrument in its composed form have not been examined. The largest limitations of the study, however, are its small sample size (*n* = 17), and the fact that only for a subset of the included patients a full set of ratings was available due to the relatively high proportion of missing data. This, together with the fact our data were highly skewed, and the risk of type-1 error due to multiple comparisons, constrained our statistical analyses. Results should therefore be interpreted with caution, and follow-up studies are extremely important.

### Implications and Suggestions for Future Research

In spite of its statistical limitations, our study provides relevant information about potential precipitants of, themes related to, and attributions given for sudden gains. The merit of this study therefore lies mainly its descriptive value. Furthermore, it laid out difficulties that one can encounter in this type of research and points toward several specific suggestions for future research. First, larger samples and more sophisticated statistical tests are needed. Second, in order to conduct a more detailed within-person analysis that focuses on proximal causes of sudden gains, one would need a more fine-grained assessment of both within- and between-session change in a larger sample of patients. Experience sampling methods (ESM) might be promising in this regard. In doing this, it might be relevant to differentiate between procedures and processes, to focus on the differentiation between the different domains, and to critically evaluate the scales of the instruments that are used. Furthermore, it is important to assess patients' attributions for change more systematically. Under ideal circumstances therapists would track sudden gains on a session-by-session basis during the study, and in case a gain is detected they would ask the client to fill out an attribution questionnaire or administer a structured interview to learn more about this. Other interesting avenues for future research might be to contrast those with and without sudden gains, especially to shed more light on the role of alliance as a prerequisite for change, and to examine whether different processes contribute to sudden gains at different time points. This might add to the generalizability and representativeness of our findings. Additionally, future research should include other potential therapy processes that are relevant to sudden gains as well, such as self-esteem (8), positive and negative life events (13), and treatment adherence (29). This might increase both generalizatility as well as representativeness of findings. To conclude, the large proportion of missing and damaged videotapes points toward the importance of optimization of procedures for in-session-recordings and video storage. In the current study, therapists used handycams with mini DV tapes that they had to set up before each session. Each tape was manually digitalized afterwards. This allows for noise in both the recording- as well as the digitalization process. Fixed, automated digital systems in which the therapist only needs to press a button to start and stop the recording and automatic encrypted upload to a secured server after the session might be helpful to solve this issue.

### To Conclude

Although the literature on sudden gains has grown substantially in the past decade, with ~100 additional studies on sudden gains published since the first meta-analysis in 2011 (3), almost none of them has focused on identifying the processes that happen in the sessions surrounding the gains. Sudden gain process research is an example of detailed time-course research. This type of research provides a powerful tool for testing mechanism hypotheses but is time-consuming and labor intensive. Probably because of this, these types of studies are rarely carried out, leaving this to be a neglected area in therapy mechanism research. Increased utilization of this approach may provide insight into the mechanisms of change in psychotherapy and thereby contribute to treatment enhancing strategies. We would therefore like to encourage other researchers to conduct follow-up research. Our study could serve as a framework for this.

## Data Availability Statement

The anonymized raw data supporting the conclusions of this article will be made available by the authors, without undue reservation.

## Ethics Statement

The study was reviewed and approved by Maastricht University's Ethical Board. The patients/participants provided their written informed consent to participate in this study.

## Author Contributions

MH and LL obtained funding for the study. LL, RD, TT, and MH designed the study. LL conducted the study and supervised the raters, in consultation with RD, TT, and MH. LL performed the data analysis, with assistance from JS-S, and drafted the manuscript. All authors contributed to the article and approved the submitted version.

## Conflict of Interest

TT was employed by Empirically Validated Investment Strategies Co. The remaining authors declare that the research was conducted in the absence of any commercial or financial relationships that could be construed as a potential conflict of interest.

## Footnotes

^1^Readers familiar with our study (14) might notice that the initial study identified 27 patients with sudden gains in CT and also included an Interpersonal Psychotherapy (IPT) arm. However, since there were insufficient IPT tapes available to make statements about precipitants of IPT and/or to compare both interventions (less SG's in IPT, many missing tapes, complete data for only two patients), we decided to only focus on CT here. Furthermore, as mentioned in the paper, for CT, 8 out of 27 patients experienced gains that occurred in between-session intervals that were more than 14 days apart. To ensure the *suddenness* of the gain, for the present study, we selected only those patients whose gains occurred within a 14-day timeframe leaving us with 19 participants. As two of them did not give consent for videotaping their sessions, this resulted in a sample of 17 patients. There were no relevant differences between the sample of excluded (*n* = 10) and included (*n* = 17) patients in terms of the sociodemographic factors, depression- and treatment specifiers, and sudden gains characteristics described in the “Data Source” section.

^2^Item selection took place as follows: first an extensive literature search was conducted to gain insight in the topics, scales and items that were used in each of the domains and in related studies. Subsequently, the selected topics and scales were discussed and carefully examined in expert meetings, and items were deducted until only the most important items remained. In doing this, attention was paid to both item content as well as the number of items per domain.
