# Supplementary material for: Therapy Processes Associated With Sudden Gains in Cognitive Therapy for Depression: Exploring Therapeutic Changes in the Sessions Surrounding the Gains
Source: Front Psychiatry. 2021 Mar 23;12:576432. doi: 10.3389/fpsyt.2021.576432 (PMC8021707; doi:10.3389/fpsyt.2021.576432)
Supplement: Supplementary file 1 [file Table_1.pdf]

INSTRUMENT FOR RATING PROCESSES RELATED TO SUDDEN GAINS: CRITICAL THERAPEUTIC EVENTS AND ATTRIBUTIONS

Instructions for raters:

- Take notes while watching the tape. Be as specific as possible in transcribing.
- Rate only progress explicitly acknowledged by the client. Never infer!
  - Example explicitly acknowledged: when a patient realized that her belief that she was unattractive because her boyfriend had left her was false she empathically declared, '*I can't believe I actually thought that! How could I let him decide if I'm attractive or not'*
  - Not explicitly related to change in the belief: the patient merely reported she is hopeful about dating other men: '*I'm think I'm ready to date other men*' (this is not an explicit change in her belief about being unattractive).
- Procedure
  1. Decide if an item is applicable (yes/no),
  2. Specify the part of the question that suits the example (e.g. behavioral change item 1: *The client acknowledges an alternative behaviour as potentially beneficial and showed a willingness to try.* Write down whether the example you rate is related to *acknowledging behavior as potentially beneficial*, or whether you rate this because the client shows a *willingness to try.* Note, sometimes it is both).
  3. Specify the situation (write down the sentence in which the client acknowledged the progress + write down the point in time, e.g. 5;30m).
  4. Determine whether the change is achieved <u>during</u> the session or <u>prior to</u> the session.
     - Within session change = therapeutic progress achieved during the session (a result of working together in the session)
     - If a client reached a new understanding before a session and comes to tell the therapist about it: prior to the session.
     - Sometimes it is hard to judge whether the client knew something beforehand or just realized a new thought in session.
     - The tapes can often be helpful in resolving this. "1" can often be given to these somewhat ambiguous situations.
     - In general, the better the quality of CT in the session, the easier it is to rate cognitive changes and determine when they occurred. (The back and forth discussion between the therapist and the client on a negative thought make it much more clear whether a cognitive change is new and how well the client has accepted it. But if the conversation if just drifting around, it is much harder to figure that out).
  5. In case change is achieved prior to the session, write down whether it was given as a reason for symptom improvement by the patient.
  6. Rate the magnitude of the progress (0 – 4). Several factors should be taken into account: including the relevance of the issue and the sincerity and enthusiasm of the client, non-verbal communication, and the fact that change comes from the patient (instead of him/her just repeating what the therapist is saying). In general:
     - 0 = item not applicable, no change
     - 1 = Ambiguous (raters notice something that is not specific enough for a higher magnitude, but also not 0).
     - 2 = Definite change: but personal significance is not emphasized by the client
     - 3 = Definite change with personal advantage (theme of treatment, client explicitly acknowledges
     - 4 = Extra ordinary change
     - Note: distinction between 3 and 4 is difficult to make. In practice: 4 are almost never rated.

Note:

- Every change should get an individual magnitude score. Even when they're in the same category. For example, if you notice two changes with regard to changes in beliefs write them both down and rate them individually (as if this would be the only one).
- Sometimes it is difficult to determine whether two activities are separate enough to deserve their own magnitude score. Rules of thumb:
    - Situations deserve their own magnitude score if the topic is different, a significant amount of time is spent on each example, and the discussion about each example ends with a specific conclusion.
    - Situations do not deserve their own magnitude score if: several examples are 'listed' without further exploration in the session (e.g. client says: *'I can try to call my friend, go for a walk, fix my car, look up information about traveling with groups, work on my family tree'*, but no further examination is done).
    - Sometimes it can be a combination of the two situations mentioned above. But only if this is explicitly acknowledged in the session as such. (e.g. patient and therapist generate a list of things the patient can try. They explicitly make a distinction between things she can do by herself, and things she can do with others. They generate several examples in each category. Because of the explicit distinction, and the 'listing' of specific examples in each category, this can be rated as two separate categories).
- Several examples fit several categories because a lot of work is done in the session. For example a patient acknowledges a 'willingness to try' something, while also 'deciding to change it', and making a 'specific plan' all in one session. This example can be rated in several categories, as long as it adds something to the previous statement. IN other words: if you could do the previous statement without doing the next, than you should rate it in the other statement again.
- If you notice an important therapeutic change that is either unacknowledged by the client, or unrateable by this scale, please write a special note describing that progress instead of using this scale.

GENERAL QUESTIONS

**Was there a discussion between therapist and client about and changes in mood?**							
No							
Yes		Was change improvement?	YES		No, change was deterioration		
		If improvement, specify					
		When did improvement occur:	In today's session		prior to session		

**Did the patient report reasons for change?**							
No		Specify	There was change, but patient did not report reasons		There was no change		
Yes		What are the patient's ideas about why he/she is feeling better/worse?					
		Main reason					
		Other reasons					

**Did the patient report on anything from the previous session that stood out for him/her?**		
No		
Yes		Specify what stood out for the patient:

During/since the last session the patient reports that he/she:

**Realized something not realized before, arrived at a new perspective on something, or changed beliefs or ideas?**		Yes		No	
If yes: specify					
**Learned new techniques that he/she found helpful?**		Yes		No	
If yes: specify					
**Learned other things**		Yes		No	
If yes: specify					
**Has noticed that he/she has been doing anything differently?**		Yes		No	
If yes: specify					

COGNITIVE CHANGE

1	**The patient became aware of the relationship between cognition and mood**						
High scores if the patient explicitly acknowledges the relation between cognition and mood, and/or gives specific examples to illustrate this. Note: in sessions were awareness about the relationship between interpersonal functioning and mood is raised because the therapist provides the interpersonal rational, the client's (spontaneous, of therapist initiated) reaction determines whether a magnitude score is given! If the client responds to the rational (e.g. by recognizing/acknowledging this pattern, or giving a specific example), or client and therapist use this information to actively work on this together, a magnitude can be given to this item. If the therapist only tells the patient about it, without further exploring this and/or the patient does not explicitly respond to this (but instead e.g. only nods), this can be rated as no change.							
No							
Yes		Specify:					
In today's session?		Explain:					
Prior to the session?		Was this given as a reason for symptom improvement?					
		Yes		No			
		Explain:					
Magnitude		0	1	2	3	4	
		No Change	Possible/Potential	Definite change	Important change	Extraordinary change	

2	**The patient became aware of a belief behind negative feelings**						
Focus on change of content. The client became aware of a belief that s/he was unaware of before, and this belief should be related to the client's distress. For example, if a client became aware that he thought about doing some shopping after therapy, and the thought is unrelated to his depression in any way, then it should not be rated here. A more appropriate example would be: a client's date did not show up last night, and she was upset but did not consciously know what thoughts triggered the negative feeling. After working with the therapist, she finally realized that she had the thought that "he did not show up because I am so ugly."							
No							
Yes		Specify:					
In today's session?		Explain:					
Prior to the session?		Was this given as a reason for symptom improvement?					
		Yes		No			
		Explain					
Magnitude		0	1	2	3	4	
		No Change	Possible/Potential	Definite change	Important change	Extraordinary change	

**3**	**The patient changed his/her belief**						
Focus on change of content. The client changed his/her beliefs. This could take several forms, including acknowledging errors in old beliefs, acknowledging errors in old thinking habits, arriving at an alternative explanation, coming to a new belief, or adopting a rational response to old beliefs. Note: Rate only progress explicitly acknowledged by the client. Never infer! Example explicitly acknowledged: when a patient realized that her belief that she was unattractive because her boyfriend had left her was false she empathically declared, 'I can't believe I actually thought that! How could I let him decide if I'm attractive or not' Not explicitly related to change in the belief: the patient merely reported she is hopeful about dating other men: 'I'm think I'm ready to date other men' (this is not an explicit change in her belief about being unattractive).							
No							
Yes		Specify:					
In today's session?		Explain:					
Prior to the session?		Was this given as a reason for symptom improvement?					
		Yes		No			
		Explain					
Magnitude		0	1	2	3	4	
		No Change	Possible/Potential	Definite change	Important change	Extraordinary change	

**4**	**The patient became aware of schema**						
Focus on change of content. Item 4 parallels items 2, although it applies to schema rather than to specific beliefs. Schemata are patterns of thinking that organize the client' life experience and form the basis for beliefs. They represent the core of the cognitive disturbance, and can be thought of as "core beliefs." Compared to specific beliefs relevant to item 2, schemata are usually much broader in scope. Here are a few examples: "If I am not perfect, then I am a failure;" "Nobody will ever love me (because I am so clumsy);" and "I am a horrible person."							
No							
Yes		Specify:					
In today's session?		Explain:					
Prior to the session?		Was this given as a reason for symptom improvement?					
		Yes		No			
		Explain					
Magnitude		0	1	2	3	4	
		No Change	Possible/Potential	Definite change	Important change	Extraordinary change	

**5**	**The patient changed his/her schema**					
Focus on change of content. Item 5 parallels item 3, although it applies to schema rather than to specific beliefs. Schemata are patterns of thinking that organize the client' life experience and form the basis for beliefs. They represent the core of the cognitive disturbance, and can be thought of as "core beliefs." Compared to specific beliefs relevant to item 3, schemata are usually much broader in scope. Here are a few examples: "If I am not perfect, then I am a failure;" "Nobody will ever love me (because I am so clumsy);" and "I am a horrible person."						
No						
Yes		Specify:				
In today's session?		Explain:				
Prior to the session?		Was this given as a reason for symptom improvement?				
		Yes		No		
		Explain				
Magnitude		0	1	2	3	4
		No Change	Possible/Potential	Definite change	Important change	Extraordinary change

**6**	**The patient accepted a new cognitive technique**					
Focus on agreeing to change the style of thinking. The client acknowledged a specific cognitive technique as potentially beneficial and showed a willingness to use it. Note: we consider structuring or scheduling daily activities as a cognitive technique too, as long as it is related to the conscious choice to take matter into own hands, and choose the activities that are good for the client. In other words: only rate planning/scheduling as a cognitive technique if the relation mood/activities is clear.						
No						
Yes		Specify:				
In today's session?		Explain:				
Prior to the session?		Was this given as a reason for symptom improvement?				
		Yes		No		
		Explain				
Magnitude		0	1	2	3	4
		No Change	Possible/Potential	Definite change	Important change	Extraordinary change

BEHAVIOURAL CHANGE

The patient accepted alternative behaviour						
Focus on agreeing to change behaviour patterns. The client acknowledges an alternative behaviour as potentially beneficial and showed a willingness to try.						
No						
Yes		Specify:				
In today's session?		Explain:				
Prior to the session?		Was this given as a reason for symptom improvement?				
		Yes		No		
		Explain				
Magnitude		0	1	2	3	4
		No Change	Possible/Potential	Definite change	Important change	Extraordinary change

The patient decided to increase pleasurable activities						
Item addresses progress made in increasing pleasurable activities for the client. Focus on decision to increase activities						
No						
Yes		Specify:				
In today's session?		Explain:				
Prior to the session?		Was this given as a reason for symptom improvement?				
		Yes		No		
		Explain				
Magnitude		0	1	2	3	4
		No Change	Possible/Potential	Definite change	Important change	Extraordinary change

**The patient made plans for pleasurable activities**							
Item addresses progress made in increasing pleasurable activities for the client. Focus on plans how to increase these activities							
No							
Yes		Specify:					
In today's session?		Explain:					
Prior to the session?		Was this given as a reason for symptom improvement?					
		Yes		No			
		Explain					
Magnitude		0	1	2	3	4	
		No Change	Possible/Potential	Definite change	Important change	Extraordinary change	

**The patient engaged in a wide and diverse array of activities**							
No							
Yes		Specify:					
In today's session?		Explain:					
Prior to the session?		Was this given as a reason for symptom improvement?					
		Yes		No			
		Explain					
Magnitude		0	1	2	3	4	
		No Change	Possible/Potential	Definite change	Important change	Extraordinary change	

**The patient structured his/her day's activities**							
No							
Yes		Specify:					
In today's session?		Explain:					
Prior to the session?		Was this given as a reason for symptom improvement?					
		Yes		No			
		Explain					
Magnitude		0	1	2	3	4	
		No Change	Possible/Potential	Definite change	Important change	Extraordinary change	

INTERPERSONAL CHANGE

Note: carefully read the wording of the items to see the subtle difference between item 1 2 3 vs. 4 5 6. Main difference is that for 1 2 and 3, the awareness of the role of interpersonal functioning has to be there in order to receive a magnitude score (aware). Items 4, 5, 6 can be rated without an explicit link to interpersonal change (decided, made plans, changed). For example question 3: the wording of the item allows a magnitude score if a patient decides to increase interpersonal functioning, even though this is mainly from an increase activities point of view instead of a conscious interpersonal link (because literally seen, the patient has decided to increase this). It has to be noted however, that this will probably be a low score.

**The patient became aware of the relation between interpersonal functioning and mood**						
Note: in sessions were awareness about the relationship between interpersonal functioning and mood is raised because the therapist provides the interpersonal rational, the client's (spontaneous, of therapist initiated) reaction determines whether a magnitude score is given! If the client responds to the rational (e.g. by recognizing/acknowledging this pattern, or giving a specific example), or client and therapist use this information to actively work on this together, a magnitude can be given to this item. If the therapist only tells the patient about it, without further exploring this and/or the patient does not explicitly respond to this (but instead e.g. only nods), this can be rated as no change.						
No						
Yes		Specify:				
In today's session?		Explain:				
Prior to the session?		Was this given as a reason for symptom improvement?				
		Yes		No		
		Explain				
Magnitude		0	1	2	3	4
		No Change	Possible/Potential	Definite change	Important change	Extraordinary change

**The patient became aware of dysfunctional patterns in interpersonal functioning**						
No						
Yes		Specify:				
In today's session?		Explain:				
Prior to the session?		Was this given as a reason for symptom improvement?				
		Yes		No		
		Explain				
Magnitude		0	1	2	3	4
		No Change	Possible/Potential	Definite change	Important change	Extraordinary change

**The patient became aware of the need to improve interpersonal functioning**						
Note: there has to be an awareness of the interpersonal component. E.g. If the client agrees with the therapist that it is nice to do things with other people, sees this mainly a behavioural thing (a general increase of activities) and does not specifically relate this to interpersonal functioning no magnitude score is given. In other words: if a client does not distinguish doing things with other from the nice feeling he get when doing things by himself, rate 0.						
No						
Yes		Specify:				
In today's session?		Explain:				
Prior to the session?		Was this given as a reason for symptom improvement?				
		Yes		No		
		Explain				
Magnitude		0	1	2	3	4
		No Change	Possible/Potential	Definite change	Important change	Extraordinary change

**The patient decided to change interpersonal functioning**						
Note: the wording of this item allows a magnitude score if a patient decides to increase interpersonal functioning, even though this is mainly from an increase activities point of view instead of a conscious interpersonal link (because literally seen, the patient has decided to increase this). It has to be noted however, that this will probably be a low score.						
No						
Yes		Specify:				
In today's session?		Explain:				
Prior to the session?		Was this given as a reason for symptom improvement?				
		Yes		No		
		Explain				
Magnitude		0	1	2	3	4
		No Change	Possible/Potential	Definite change	Important change	Extraordinary change

**The patient made plans for changing interpersonal functioning**							
Note: the wording of this item allows a magnitude score if a patient makes plans to increase interpersonal functioning, even though this is mainly from an increase activities point of view instead of a conscious interpersonal link (because literally seen, the patient did make plans). It has to be noted however, that this will probably be a low score.							
No							
Yes		Specify:					
In today's session?		Explain:					
Prior to the session?		Was this given as a reason for symptom improvement?					
		Yes		No			
		Explain					
Magnitude		0	1	2	3	4	
		No Change	Possible/Potential	Definite change	Important change	Extraordinary change	

**The patient changed interpersonal functioning**							
Note: the wording of this item allows a magnitude score if a patient changed interpersonal functioning, even though this is mainly from an increase activities point of view instead of a conscious interpersonal link (because literally seen, the patient did change it). It has to be noted however, that this will probably be a low score.							
No							
Yes		Specify:					
In today's session?		Explain:					
Prior to the session?		Was this given as a reason for symptom improvement?					
		Yes		No			
		Explain					
Magnitude		0	1	2	3	4	
		No Change	Possible/Potential	Definite change	Important change	Extraordinary change	

ALLIANCE

- WAI-O-S (Bordin) → 12 items, measuring Bond, Goal, Task

CT		IPT		rating code	

Rater ID, Tape ID

Working Alliance Inventory Form O -- Short Version

Patient #		Center		Rater Name	
Session #		Therapist		Rating Date	

Instructions -- On this page, there are 12 sentences that describe some of the different ways a therapist/client dyad may interact in therapy. If a statement describes the way you always (consistently) perceive the dyad, circle the number 6; if it never applies to the dyad, circle the number 0. Use the numbers in between to describe the variations between these extremes.

This questionnaire is CONFIDENTIAL; neither the therapist, client, nor the agency will see your answers. Work fast, your first impressions are the ones we would like to see. (PLEASE DON'T FORGET TO RESPOND TO EVERY ITEM.)

A. O. Horvath, 1981 1982, V. Tichenor 1989, A. O. Horvath, 1990

1	2	3	4	5	6	7
Never	Rarely	Occasionally	Sometimes	Often	Very Often	Always

1. There is agreement about the steps taken to help improve the client's situation. ______
2. There is agreement about the usefulness of the current activity in therapy (i.e., the client is seeing new ways to look at his/her problem). ______
3. There is a mutual liking between the client and therapist. ______
4. There are doubts or a lack of understanding about what participants are trying to accomplish in therapy. ______
5. The client feels confident in the therapist's ability to help the client. ______
6. The client and therapist are working on mutually agreed upon goals. ______
7. The client feels that the therapist appreciates him/her as a person. ______
8. There is agreement on what is important for the client to work on. ______
9. There is mutual trust between the client and therapist. ______
10. The client and therapist have different ideas about what the client's real problems are. ______
11. The client and therapist have established a good understanding of the changes that would be good for the client. ______
12. The client believes that the way they are working with his/her problem is correct. ______
